# Genetic modification to induce CXCR2 overexpression in mesenchymal stem cells enhances treatment benefits in radiation-induced oral mucositis

**DOI:** 10.1038/s41419-018-0310-x

**Published:** 2018-02-14

**Authors:** Zongshan Shen, Jiancheng Wang, Qiting Huang, Yue Shi, Zhewei Wei, Xiaoran Zhang, Yuan Qiu, Min Zhang, Yi Wang, Wei Qin, Shuheng Huang, Yinong Huang, Xin Liu, Kai Xia, Xinchun Zhang, Zhengmei Lin

**Affiliations:** 1 Guangdong Provincial Key Laboratory of Stomatology, Department of Operative Dentistry and Endodontics, Guanghua School of Stomatology, Sun Yat-sen University, Guangzhou, China; 2 The Key Laboratory for Stem Cells and Tissue Engineering, Center for Stem Cell Biology and Tissue Engineering, Ministry of Education, Sun Yat-sen University, Guangzhou, China; 3 Department of Gastrointestinal Surgery of the First Affiliated Hospital of Sun Yat-sen University, Guangzhou, China; 4 Department of Andrology, The First Affiliated Hospital, Sun Yat-sen University, Guangzhou, China; 5 Department of Prosthodontics, Hospital of Stomatology, Institute of Stomatological Research, Guanghua School of Stomatology, Sun Yat-sen University, Guangzhou, China

## Abstract

Radiation-induced oral mucositis affects patient quality of life and reduces tolerance to cancer therapy. Unfortunately, traditional treatments are insufficient for the treatment of mucositis and might elicit severe side effects. Due to their immunomodulatory and anti-inflammatory properties, the transplantation of mesenchymal stem cells (MSCs) is a potential therapeutic strategy for mucositis. However, systemically infused MSCs rarely reach inflamed sites, impacting their clinical efficacy. Previous studies have demonstrated that chemokine axes play an important role in MSC targeting. By systematically evaluating the expression patterns of chemokines in radiation/chemical-induced oral mucositis, we found that CXCL2 was highly expressed, whereas cultured MSCs negligibly express the CXCL2 receptor CXCR2. Thus, we explored the potential therapeutic benefits of the transplantation of CXCR_2_-overexpressing MSCs (MSCs^CXCR2^) for mucositis treatment. Indeed, MSCs^CXCR2^ exhibited enhanced targeting ability to the inflamed mucosa in radiation/chemical-induced oral mucositis mouse models. Furthermore, we found that MSC^CXCR2^ transplantation accelerated ulcer healing by suppressing the production of pro-inflammatory chemokines and radiogenic reactive oxygen species (ROS). Altogether, these findings indicate that CXCR2 overexpression in MSCs accelerates ulcer healing, providing new insights into cell-based therapy for radiation/chemical-induced oral mucositis.

## Introduction

Approximately 80–100% of patients with head and neck cancers who receive radiation treatment develop oral mucositis, which is the most common complication of this treatment^1^. Oral mucositis affects food intake and swallowing and speaking ability, ultimately leading to malnutrition, and can lead to life-threatening bacteremia^2,3^, thereby reducing patient tolerance to cancer therapy and patient survival^3^. Previous studies have found that oxidative stress induced by radiation leads to reactive oxygen species (ROS) production, which greatly impacts mucositis because ROS damage DNA, induce cell apoptosis, and increase pro-inflammatory cytokine release^4^. However, traditional treatments, such as pain management, nutrition support therapy, and antibiotics administration, can alleviate the symptoms of mucositis but are not sufficient for the prevention or treatment of this condition^1,4,5^. Moreover, these treatments elicit severe side effects, such as opportunistic infections and lipid metabolic disorder. Therefore, it is essential to explore effective treatments with fewer adverse effects.

Because mesenchymal stem cells (MSCs) exhibit beneficial immunomodulatory, anti-oxidative, and anti-inflammatory characteristics, MSC therapy has been reported to be effective for patients with a series of inflammatory and radiogenic diseases, including myocardial infarction (MI), spinal cord injury, osteomyelitis, Crohn’s disease, and radiogenic skin inflammation^6–9^. These studies indicated that MSC transplantation might represent a promising therapy for radiogenic mucositis. In a clinical setting, MSCs are typically administered through two routes: local transplantation and systemic infusion. Because radiogenic mucositis is distributed in various parts of the human body, local transplantation is not appropriate. Additionally, local implantation has many limitations, such as significant morbidity and disruption of the structure of the local environment^10^. Thus, intravascular administration is much more appropriate. However, the low migratory efficiency of MSCs into the inflamed mucosa limits this approach and reduces its clinical benefits^11^. Therefore, studies aimed at promoting MSC migration toward mucositis sites are vital.

Chemokine axes control the migratory patterns of MSCs to specific sites (i.e., injured sites)^12,13^. Chemokines released from inflammatory tissues might activate adhesion ligands and promote the transendothelial migration or subsequent implantation of MSCs in the surrounding tissues^14^. The targeting of MSCs toward inflamed sites relies on specific chemokine receptors. However, the expression of these receptors in MSCs decreases after in vitro expansion^15^. To enhance their migratory ability, researchers have attempted to overexpress the corresponding receptors in MSCs. In our previous study, CXCR5-overexpressing MSCs exhibited enhanced targeting ability to the inflamed skin in a contact hypersensitivity (CHS) mouse model, in which CXCL13 was notably upregulated. Moreover, these genetically modified MSCs with enhanced targeting ability markedly suppress skin inflammation^13^. Therefore, methods that re-establish the interactions between tissue-specific chemokines and their corresponding receptors on MSCs are promising strategies for enhancing the targeting ability of MSCs and thereby improve the therapeutic benefits of MSC therapy.

Here, overexpression of the chemokine receptor CXCR2 on MSCs improved cell migration to the inflamed mucosa and promoted cell survival in oral radiation/chemical-induced mucositis (RIM/CIM). Furthermore, CXCR2-overexpressing MSCs (MSCs^CXCR2^) accelerated ulcer healing, likely by suppressing ROS and pro-inflammatory chemokine production. Thus, this innovative strategy that enhances the therapeutic benefits shows promise for future clinical applications.

## Results

### CXCL2 is upregulated in radiation/chemical-induced oral mucositis

**Fig. 1:**
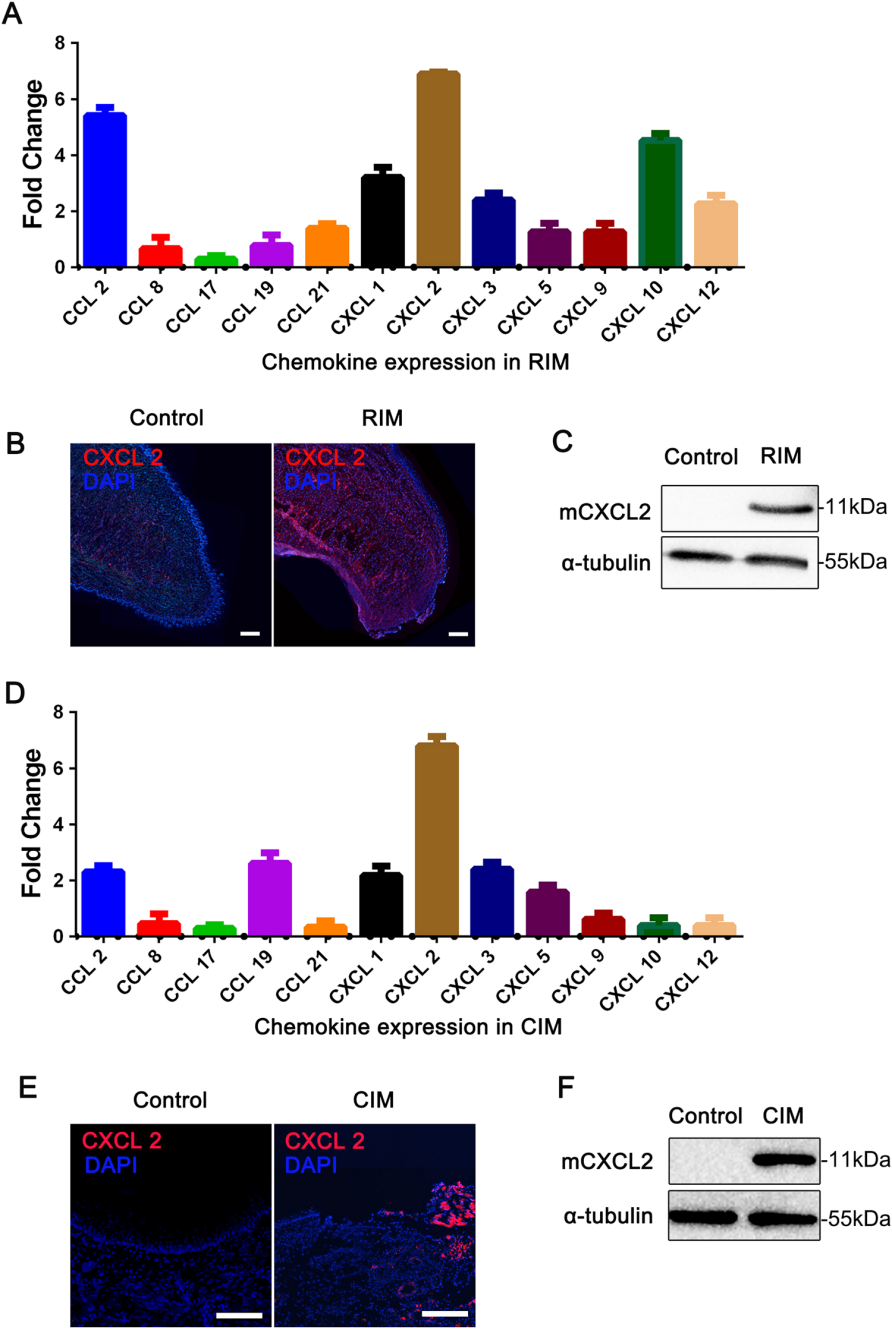
CXCL2 is upregulated in radiation/chemical-induced mucositis. **a** The mRNA expression levels of various chemokines involved in radiation-induced tongue mucositis were analyzed by RT-qPCR. Samples were extracted from normal and inflamed tongues. The fold change represents the expression of each chemokine mRNA in inflamed tongues compared with normal tongues on day 7 after radiation. All the data are presented as the means ± standard errors of the means (SEMs) (*n* = 6) for each group. **b** Immunofluorescence staining of CXCL2 (red) in normal and inflamed tongues on day 5 after radiation. The experiments were repeated three times in individual mice. Nuclei were visualized through DAPI staining (blue). Scale bar = 100 μm. **c** Total tissue lysates of normal and inflamed tongues were subjected to western blot analysis of mouse CXCL2 protein expression. The experiments were repeated three times, and a representative blot is shown. **d** The fold changes in the mRNA levels of various chemokines in chemical-induced mucositis were analyzed by RT-qPCR. Samples were extracted from normal and inflamed mucosal tissues on day 5 after chemical stimulation. The data are presented as the means ± SEMs (*n* = 6) for each group. **e** Immunofluorescence staining for CXCL2 (red) in normal and inflamed mucosal tissues on day 5 after chemical stimulation. The experiments were repeated three times in individual mice. Nuclei were visualized through DAPI staining (blue). Scale bar = 100 μm. **f** Total tissue lysates from normal and inflamed mucosal tissues were subjected to western blot analysis of mouse CXCL2. RIM radiation-induced mucositis, CIM chemical-induced mucositis

To systematically investigate the expression of chemokines during the inflammatory phase of RIM/CIM, we evaluated the mRNA expression of chemokines associated with skin and mucosal inflammation, including CCL2, CCL8, CCL17, CCL19, CCL21, CXCL1, CXCL2, CXCL3, CXCL5, CXCL9, CXCL10, and CXCL12^16–19^. We found that the mRNA levels of various CXCR2 ligands, including CXCL1, CXCL3, CXCL5, and CXCL2, were upregulated. The CXCL2 mRNA levels were markedly upregulated after radiation compared with normal tissues (Fig. 1a). Furthermore, CXCL2 upregulation was confirmed by in situ immunofluorescence staining and western blotting (Fig. 1b, c). Interestingly, the expression of CXCL2 mRNA peaked on day 7 after radiation and then gradually declined (Supplementary Fig. 2A), which was consistent with the clinical symptoms. CIM is another model applied for studying oral mucositis caused by cancer therapy^20^. Similarly, the expression levels of CXCL2 mRNA and protein were substantially increased in CIM (Fig. 1d–f). However, the highest levels of CXCL2 mRNA expression were observed 5 days after chemical induction (Supplementary Fig. 2B). These results indicate that CXCL2 is the dominant chemokine expressed in oral mucositis.

### CXCR2-overexpressing MSCs retain the characteristics of human MSCs

**Fig. 2:**
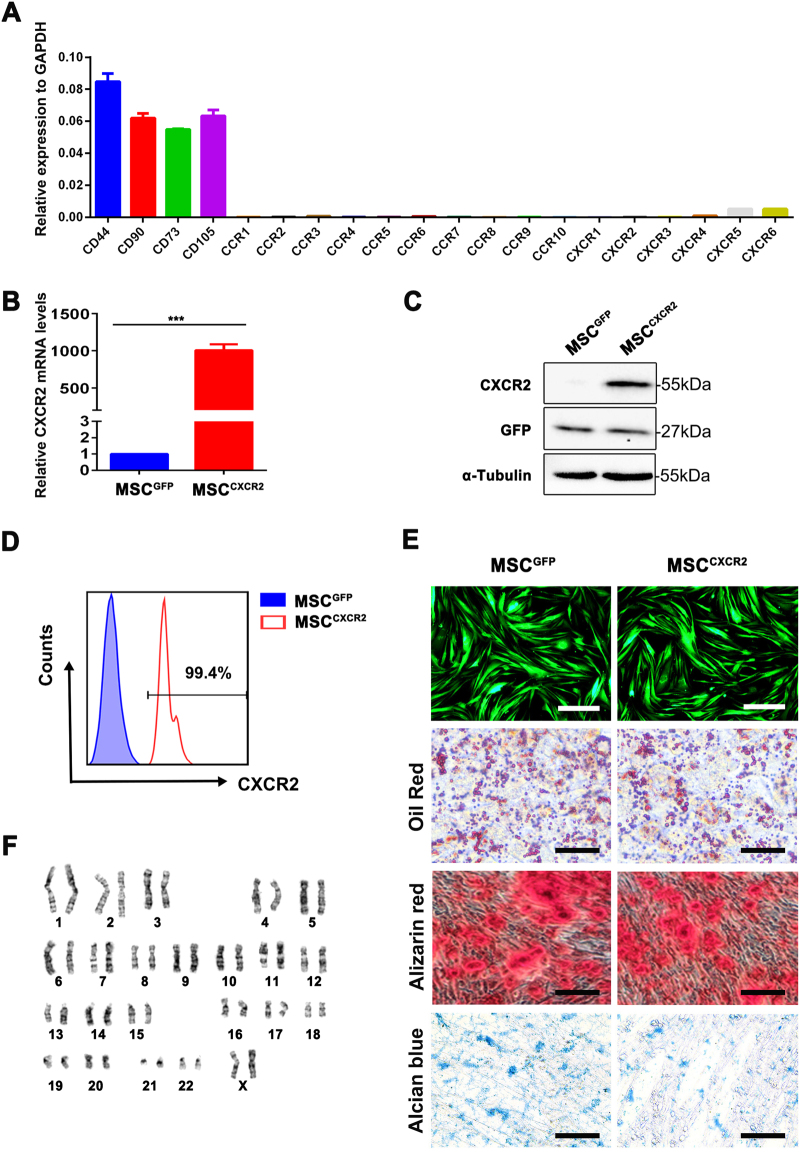
CXCR2-overexpressing MSCs retain the characteristics of human MSCs. **a** Histograms representing the mRNA expression levels of C–C and C–X–C chemokine receptors from three independent samples of sixth-passage MSCs. MSC surface markers (CD44, CD90, CD73, and CD105) were detected as a positive control. The data are presented as the means ± SEMs for each group (*n* = 3, *t*-test). **b** The expression levels of CXCR2 mRNA in MSCs^GFP^ and MSCs^CXCR2^ after gene transduction were analyzed via RT-qPCR. Glyceraldehyde 3-phosphate dehydrogenase (GAPDH) mRNA was detected as an internal control. The data are presented as the means ± SEMs for each group (*n* = 3, *t*-test). **c** Total MSC^GFP^ and MSC^CXCR2^ lysates from three independent samples were subjected to western blot analysis of CXCL2 expression. **d** A flow cytometry analysis was used to detect CXCR2 protein on the surface of MSCs^GFP^ and MSCs^CXCR2^. Six independent samples were analyzed. **e** Representative images showing the trilineage differentiation potential of MSCs^CXCR2^ and MSCs^GFP^ into adipocytes (oil red O), osteocytes (alizarin red), and chondrocytes (alcian blue). Scale bar = 20 µm. **f** Cytogenetic analysis of MSCs^CXCR2^ at the eighth passage. The experiments were repeated three times

Chemokine receptors play an essential role in MSC targeting to inflamed sites by binding to their corresponding chemokines^12,21^. Therefore, we evaluated the expression of chemokine receptors on MSCs. A transcription quantitative real-time PCR (RT-qPCR) analysis demonstrated that cultured MSCs at passage six expressed very low levels of chemokine receptors, including CXCR2 (Fig. 2a; Supplementary Fig. 2c), which is the receptor of the chemokine that is highly expressed in mucositis, CXCL2.

Subsequently, we overexpressed CXCR2 in MSCs by infecting them with a lentiviral vector encoding CXCR2 to construct MSCs^CXCR2^ (Supplementary Fig. 3A) and potentially enhance the targeting of MSCs to inflamed sites. Moreover, we infected MSCs with a lentiviral vector encoding GFP (referred to as MSCs^GFP^) to allow the tracing of MSCs after transplantation (Supplementary Fig. 3B). Indeed, the CXCR2 expression levels were significantly upregulated 2 days after infection (Fig. 2b). Moreover, this upregulation was further confirmed by western blotting and flow cytometry (Fig. 2c, d). To determine whether CXCR2-overexpressing MSCs retain their identities, we investigated the expression of MSC-specific markers and the multilineage differentiation potential of the transduced MSCs. A flow cytometry analysis indicated that MSCs^CXCR2^ expressed the same pattern of stem cell surface markers as MSCs^GFP^ (Supplementary Fig. 3C). Moreover, under mesenchymal lineage differentiation conditions, both MSCs^CXCR2^ and MSCs^GFP^ showed similar trilineage differentiation potential and exhibited osteogenic, adipogenic, and chondrogenic lineage phenotypes during the 2-to-4-week differentiation period (Fig. 2e). A cytogenetic analysis demonstrated that all MSCs^CXCR2^ (at passage eight) had a normal diploid chromosomal complement (Fig. 2f). Taken together, these results indicate that the transgenic modification of MSCs does not alter the intrinsic characteristics of MSCs.

### MSCs^CXCR2^ exhibit enhanced migration potential in vitro and in vivo

**Fig. 3:**
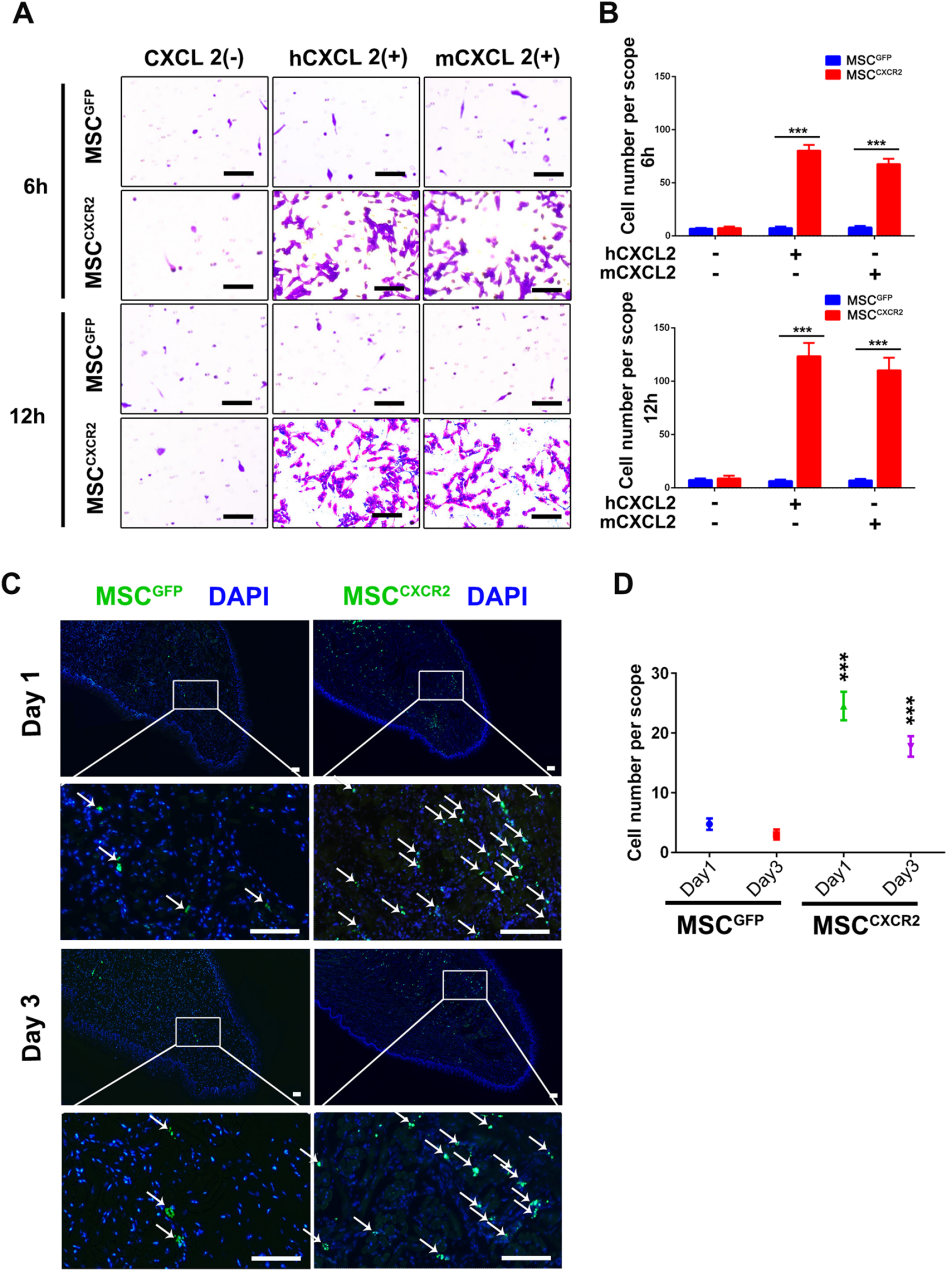
MSCs^CXCR2^ exhibit enhanced migration potential in vitro and in vivo. **a** In vitro migration of MSCs^CXCR2^and MSCs^GFP^ toward human CXCL2 (hCXCL2) or murine CXCL2 (mCXCL2). Transwell filters were stained with 0.1% crystal violet and observed under a microscope. Scale bar = 25 μm. **b** The migrated MSCs after incubation for either 6 or 12 h were quantified in each microscopic field; ***P < 0.001. **c** MSCs^CXCR2^ and MSCs^GFP^, both of which expressed GFP when injected into radiation-induced mucositis models, were examined by immunofluorescence staining on days 1 and 3 post-injection. Signals: GFP, green; DAPI, blue. Scale bar = 100 μm. **d** GFP-positive cells were quantified in each microscopic field of the mouse tongue. The data are presented as the means ± SEMs for each group (*n* = 6, *t*-test). ***P < 0.001 for the RIM + MSC^CXCR2^ group compared with the RIM + MSC^GFP^ group on the indicated day

Chemokine axes mediate MSC migration to inflamed sites^12,21^. Hence, we used a chemotaxis assay to assess whether CXCR2-overexpressing MSCs exhibited enhanced targeting ability in vitro. The results showed that MSCs^CXCR2^, but not MSCs^GFP^, strongly responded to 5 ng/ml human CXCL2 (hCXCL2) and 50 ng/ml murine CXCL2 (mCXCL2) (Fig. 3a). Additionally, the number of migrated MSCs^CXCR2^ increased nearly two-fold when the incubation time was extended from 6 to 12 h (Fig. 3b).

To determine whether MSCs^CXCR2^ had an enhanced ability to target radiation-damaged tongues in vivo, we injected MSCs^CXCR2^ and MSCs^GFP^ into RIM model mice through the tail vein on day 7 after radiation. Inflamed tongues were collected from each group on days 1 and 3 post-injection. An immunofluorescence staining analysis demonstrated that MSCs^CXCR2^ accumulated in the mucositis region in the RIM model. Interestingly, within 3 days of injection, the number of MSCs^GFP^ declined sharply, and the number of MSCs^CXCR2^ decreased slightly (Fig. 3c, d). To further investigate whether MSCs^CXCR2^ displayed enhanced targeting ability to CIM sites, we injected MSCs^CXCR2^ and MSCs^GFP^ into CIM model mice. An immunostaining analysis indicated that the number of MSCs^CXCR2^ was significantly higher than that of MSCs^GFP^ in the inflamed mucosa (Supplementary Fig. 4A, B). Altogether, these results indicate that CXCR2 overexpression enhances MSC targeting to RIM/CIM sites.

### MSCs^CXCR2^ exhibit enhanced survival in RIM

**Fig. 4:**
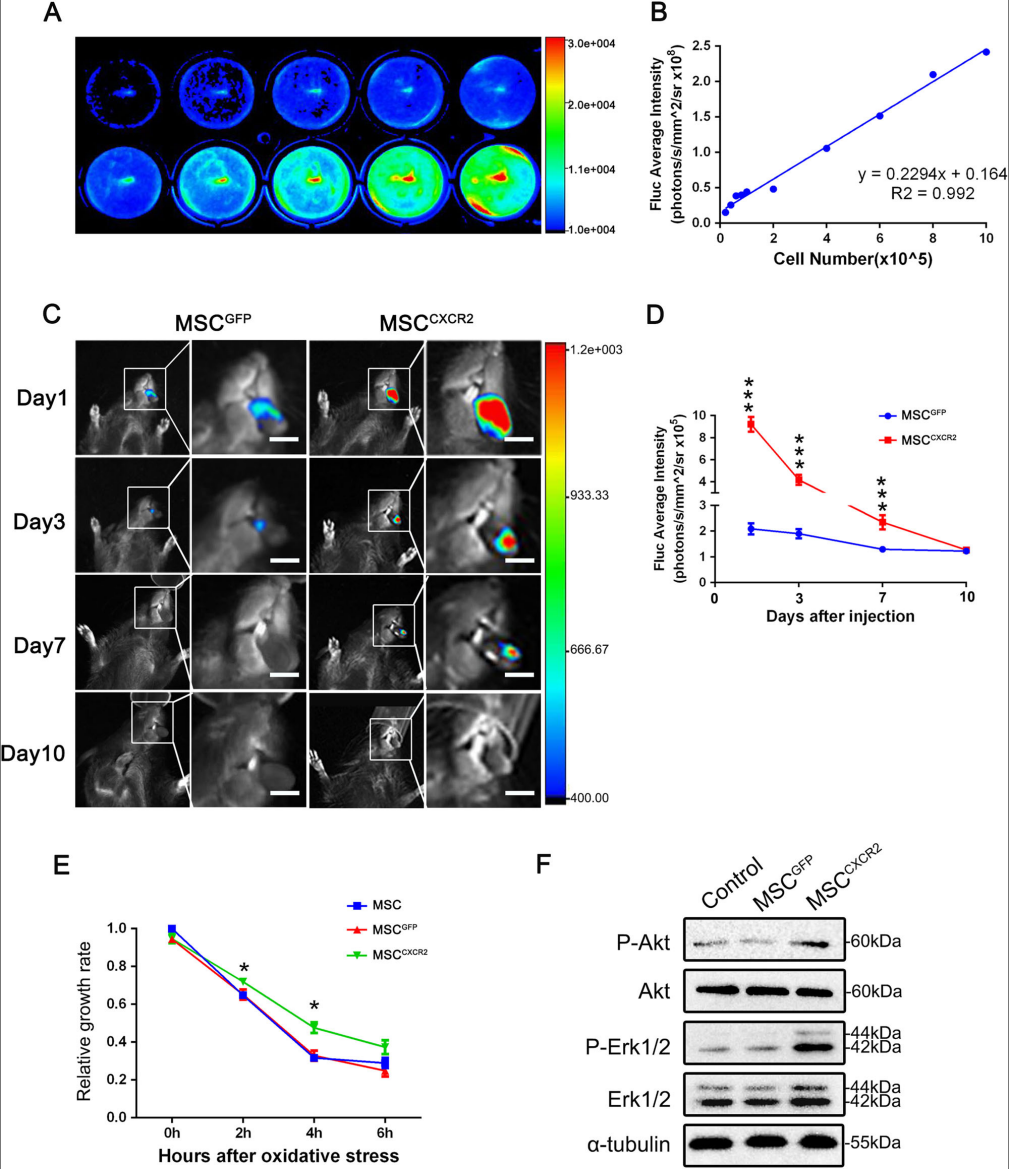
MSCs^CXCR2^ exhibit enhanced cell survival in radiation-induced mucositis. **a** In vitro bioluminescence imaging (BLI) of MSCs^CXCR2^ from groups with different cell numbers. The color scale bar represents the optical fluorescence intensity in photons/second/cm^2^/steradian (photons/s/cm^2^/sr). **b** Linear correlation of the cell number with the luc-induced luminescence intensity (*r*^2^ = 0.992). **c** In vivo BLI representing MSC survival on days 1, 3, 7, and 10 after injection. The luminescence intensity represents the number of living cells. Scale bar = 10 mm. **d** Quantitative analysis of BLI on days 1, 3, 7, and 10 after injection. The data are presented as the means ± SEMs (*n* = 3). ***P < 0.001. **e** CCK-8 assay showing the viability of MSCs^CXCR2^ and MSCs^GFP^ 2 h and 4 h after exposure to oxidative stress. The data are presented as the means ± SEMs (*n* = 3) for each group and are representative of three independent experiments. *P < 0.05. **f** Total MSC^GFP^ and MSC^CXCR2^ lysates were subjected to western blot analysis of Akt and Erk1/2 phosphorylation. The experiments were repeated three times

We inserted luciferase 2-monooxygenenase (luc) after the chemokine receptor in a lentiviral vector, placing both constructs under the control of the same promoter (Supplementary Figs. 3A, B), and infected MSCs to allow their tracing after in vivo transplantation. Bioluminescence imaging (BLI) revealed a strong linear relationship (*r*^2^ = 0.992) between the number of MSCs and the average luminescence intensity (Fig. 4a, b), indicating that the number of MSCs can be determined by the luminescence intensity. Thus, to investigate MSC survival after systemic injection in mouse models, we examined the average luminescence intensity through BLI. The average luminescence intensity of both groups peaked 1 day after infusion. Although the luminescence intensity in the MSC^GFP^ group decreased sharply within 3 days, the luminescence intensity in the MSC^CXCR2^ group remained steady for 3 days and was even observed after 7 days (Fig. 4c, d). These findings indicated that the overexpression of CXCR2 in MSCs not only enhanced their targeting ability to mucositis sites but also increased their survival rate.

To further verify that the overexpression of CXCR2 in MSCs increased their survival rate, we used an in vitro hydrogen peroxide (H_2_O_2_) model as previously reported^22^. Cell Counting Kit-8 (CCK-8) assays indicated that MSCs^CXCR2^ exhibited increased survival rates 2 and 4 h after exposure to oxidative stress compared with MSCs^GFP^ (*P* < 0.05) (Fig. 4e). The PI3K/Akt and MAPK/Erk signaling pathways have a large impact on cell survival and proliferation^23^. A western blot analysis indicated that the P-Akt and P-Erk1/2 levels were significantly increased in MSCs^CXCR2^ (Fig. 4f). These results indicated that CXCR2 enhances cell survival and proliferation, likely by activating the PI3K/Akt and MAPK/Erk signaling pathways.

### MSCs^CXCR2^ notably attenuate RIM/CIM

**Fig. 5:**
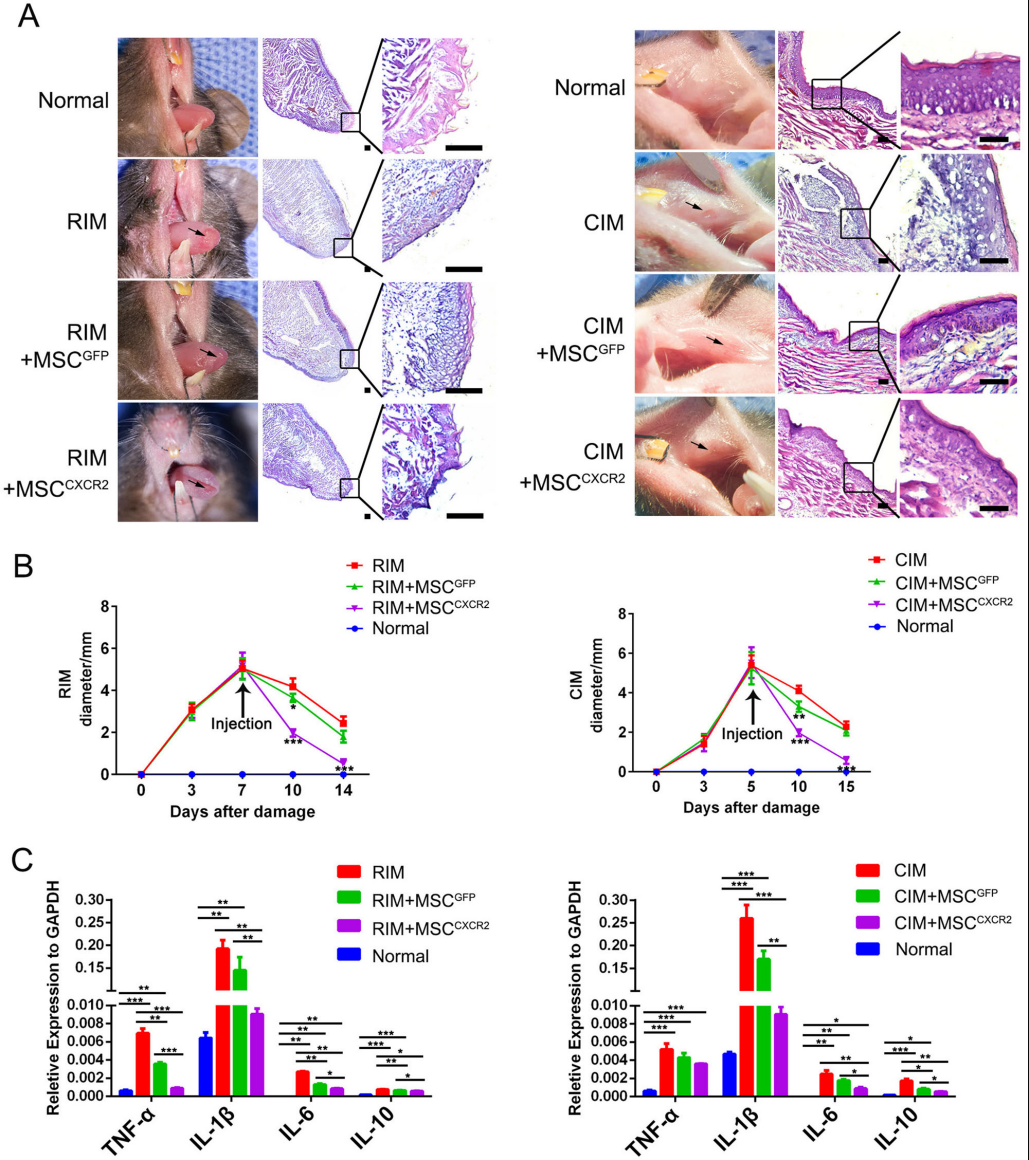
MSCs^CXCR2^ markedly attenuate radiation/chemical-induced mucositis. **a** Hematoxylin and eosin (HE)-stained tongue samples from four groups: the normal group, the RIM/CIM group, the RIM/CIM + MSC^GFP^ group, and the RIM/CIM + MSCs^CXCR2^ group. Samples were collected at 72 h post-injection, cryosectioned, and stained with HE. All the experiments were repeated three times. Scale bar = 100 μm. **b** The maximum ulcer dimension in each group was examined with Vernier calipers. The data are presented as the means ± SEMs for individual mice; *n* = 6 mice/group from three independent experiments. *P < 0.05; **P < 0.01; ***P < 0.001. **c** The levels of TNF-α, IL-1β, IL-6, and IL-10 mRNA were analyzed by RT-qPCR. mRNA samples were extracted from the tongues of mice in each group at day 3 post-injection. GAPDH mRNA was detected as an internal control. The results are representative of three experiments; *P < 0.05; **P < 0.01; ***P < 0.001

To investigate whether the enhanced migration of MSCs^CXCR2^ would improve their beneficial effect on oral mucositis, we injected mice with MSCs^GFP^ and MSCs^CXCR2^ on days 7 and 5 after radiation and chemical induction, respectively. Hematoxylin and eosin (HE) staining revealed that the group with radiation-induced ulcers exhibited a loss of filiform papillae, ulcerations, a disrupted epithelium layer, inflammatory cell infiltration, and decreased mucosal thickness. The MSC^GFP^ group showed an increase in filiform papillae and decreased ulceration, but inflammatory cell infiltration was still observed in the submucosal area. However, extensive ulceration was absent in the MSC^CXCR2^ group, which exhibited the lowest level of inflammatory cell infiltration and increased thickness of the epithelium layer and mucosal lining (Fig. 5a). We then examined the maximum diameter of ulceration in RIM/CIM models to assess the severity of mucositis. Smaller ulceration diameters in the mouse mucosa were measured in the MSC^CXCR2^ group than in MSC^GFP^ group (Fig. 5b). Inflammatory cytokines, including tumor necrosis factor-α (TNF-α), interleukin-1β (IL-1β), IL-6, and IL-10, play important roles in wound repair^4,16^. Hence, we examined the levels of inflammatory cytokines after MSC treatment to assess the anti-inflammatory effects of MSCs. A RT-qPCR analysis demonstrated that the mRNA expression of pro-inflammatory cytokines in the mouse mucosa was significantly downregulated in the MSC^CXCR2^ group but only slightly reduced in the MSC^GFP^ group (Fig. 5c). These results indicate that MSCs^CXCR2^ can markedly attenuate oral mucositis, in part due to their anti-inflammatory effects.

### MSCs accelerate mucositis recovery by decreasing radiation-induced ROS production

**Fig. 6:**
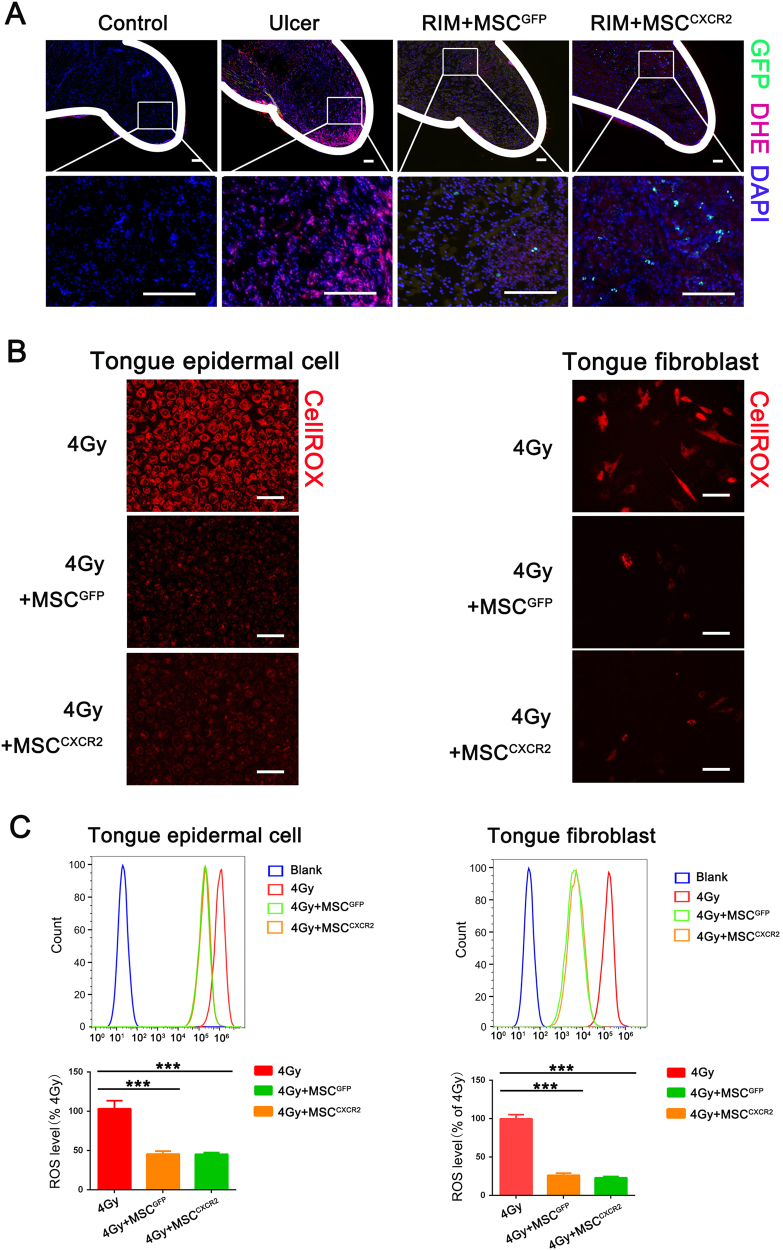
MSCs accelerate mucositis recovery by decreasing radiation-induced ROS production. **a** Representative images of DHE staining. Samples were collected on day 3 post-injection, *n* = 6 mice/group from three independent experiments. The tissue ROS levels were detected through DHE staining (red). Signals: GFP, green; DAPI, blue. Scale bar = 100 μm. **b** Primary tongue epithelial cells and tongue fibroblasts were exposed to 4 Gy of radiation and then cultured with or without MSCs for 48 h. The experiments were repeated three times. The cellular ROS levels were visualized with CellROX staining (red). Scale bar = 100 μm. **c** Density plot of the total ROS levels in primary tongue epithelial cells and tongue fibroblasts that were mono-cultured in suspension or co-cultured with MSCs in transwell chambers. The data are presented as the means ± SEMs (*n* = 3) for each group (***P < 0.001; *t*-test)

RIM is initially caused by radiogenic ROS^4,24^. Because MSCs have great anti-oxidation potential^25,26^, we wondered whether MSCs promoted ulcer healing by protecting tissue cells from ROS-induced damage. The staining of inflamed tongue tissues with the superoxide-sensitive fluorescent dye dihydroethidium (DHE) revealed that both MSC^GFP^ and MSC^CXCR2^ treatment exerted benefits by reducing the cellular ROS levels. However, MSC^CXCR2^ treatment had the greatest effect on decreasing the cellular ROS levels among all groups in vivo (Fig. 6a). We further applied CellROX^TM^ for detection of the radiogenic ROS levels in primary tongue epidermal and fibroblast cells after treatment with 4 Gy of radiation in vitro. Based on immunofluorescence staining, the radiogenic ROS levels in primary tongue cells were attenuated by co-culture with MSCs^CXCR2^ or MSCs^GFP^ (*P* < 0.05) (Fig. 6b). Furthermore, a flow cytometry analysis of the intracellular redox status confirmed that primary tongue epidermal or fibroblast cells co-cultured with MSCs exhibited less intracellular ROS generation than mono-cultured cells (Fig. 6c). In addition, an oxidative environment contributes to cell death, which is also responsible for ulcer healing delays^22,27,28^. Therefore, we used an H_2_O_2_-induced cell death model to determine whether MSCs protect tongue epidermal or fibroblast cells from cell death in an oxidative environment. Cell apoptosis was measured via Annexin V/propidium iodide (PI) staining and flow cytometry. The apoptosis of primary tongue cells subjected to co-culture with MSCs was significantly decreased compared with mono-cultured MSCs (Supplementary Fig. 5A, B). Taken together, these findings indicate that MSCs accelerate mucositis recovery, likely by decreasing radiogenic ROS production and protecting tongue cells from cell death.

## Discussion

Oral RIM is one of the most common complications of radiotherapy among patients with head and neck cancer. However, traditional treatments for severe mucositis are limited and cannot prevent ulcer recurrence. Here, we report an innovate and highly efficient treatment, namely, the systematic transplantation of MSCs with an enhanced targeting capacity to mucositis sites. This study sheds new light on the treatment of RIM and provides groundwork for the clinical application of MSC-based therapy for mucositis.

Due to their anti-inflammatory and immunomodulatory capabilities, MSCs have therapeutic potential for the treatment of inflammatory diseases^6,9^. However, systemically infused MSCs rarely home to inflamed mucosa in the orofacial region. MSC targeting depends on the interactions between chemokine receptors and specific chemokines from inflamed sites^12,21^. A RT-qPCR analysis revealed that CXCL2 was the most highly expressed chemokine in the inflamed mucosa in oral RIM mouse models. Recent studies have reported that the overexpression of corresponding chemokine receptors in MSCs can enhance their targeting ability to inflamed sites. For example, CCR1, CXCR4, and CCR7 overexpression has been shown to enhance MSC targeting to injured myocardium^29^, ischemic myocardium^30^, and secondary lymphoid organs^31^, respectively. Notably, our chemotaxis assays demonstrated that MSCs overexpressing CXCR2 had enhanced migratory ability when exposed to CXCL2 in vitro. Furthermore, immunostaining and BLI analyses revealed that MSCs^CXCR2^ exhibited enhanced targeting to the inflamed mucosa. Interestingly, we also observed that CXCR2 overexpression prolonged MSC survival in the inflamed mucosa, and this effect is likely mediated by P-Akt and P-Erk1/2 upregulation.

Subsequently, we exploited the therapeutic potential of MSC^CXCR2^ transplantation. A HE staining analysis demonstrated that mice that received MSCs^CXCR2^ displayed enhanced epithelial integrity in the mucosa compared with those that received MSCs^GFP^. Furthermore, the ulceration diameters in the MSC^CXCR2^-treated group were significantly smaller than those in the MSC^GFP^-treated control group. Together, these results suggest that MSC^CXCR2^ transplantation accelerates ulcer recovery. Conventional treatments, such as pain management and nutrition support therapy, have limited healing benefits in mucositis^1,32^. Additionally, due to conflicting evidence, no specific guideline regarding anti-inflammatory agents for RIM can be found among the Multinational Association of Supportive Care in Cancer (MASCC) clinical practice guidelines^1,5^. Recently, growth factors have been applied for the prevention of oral RIM, such as FDA-approved palfermin (recombinant human KGF)^1,33^. These factors play a marked role in the proliferation of various cell types, including endothelial cells, fibroblasts, vascular smooth muscle cells, and keratinocytes, thus promoting mucosa recovery^34^. However, because growth factors also promote angiogenesis in tumors^35,36^, the administration of growth factors might increase the risk of head and neck cancer recurrence, which hinders their application. In addition, prolonged pro-inflammatory cytokine production greatly impacts the development, metastasis, and recurrence of cancer^37^. However, growth factors can hardly alleviate mucosal inflammation. Interestingly, we found that implanted MSCs downregulated pro-inflammatory cytokines, including TNF-α, IL-1β, and IL-6. We further found that MSCs significantly decreased the cellular ROS levels both in vivo and in vitro. ROS generation is widely acknowledged to be the primary event in most mucositis-causing pathways^4,24^. For example, ROS lead to DNA damage, cell death, and upregulation of various inflammation-related signaling pathways^4,22^. Hence, the effects of MSCs on alleviating inflammation in mucositis are partially due to their anti-oxidant capability.

The mechanism of how transplanted MSCs promote tissue repair have long been debated^38–40^. MSCs accelerate tissue repair probably in three ways: by direct differentiation^38^, direct cellular interactions^39^, or secretion of soluble factors^40^. Our BLI analysis showed that MSCs were not detected in the newly formed epithelium layer and mucosal lining 10 days after injection in the RIM mouse models, suggesting that MSCs may not accelerate ulcer healing via directly generating tongue epithelial cells or fibroblast cells. In addition, we found that MSCs significantly decreased the ROS levels of tongue epithelial cell or those of the fibroblast cell in both transwell and direct co-culture systems with no significant difference between the two co-culture systems (data not show). Because MSCs can secrete factors in both co-culture systems while exhibited similar anti-oxidant effects in the two systems, MSCs might decrease the cellular ROS levels not via direct cellular interactions. Growing evidences indicate that MSCs can produce soluble factors, especially antioxidant factors, to promote tissue repair via their secretome^40,41^. Our study showed that tongue epithelial cells or fibroblast cells exhibited reduced cellular ROS levels when co-cultured with MSCs via a transwell system. We deduced that the anti-oxidative capability of MSCs mainly lies in their secretion of factors. However, these anti-oxidative secretion factors of MSCs needs to be further identified.

In summary, the transplantation of CXCL2-overexpressing MSCs promotes ulcer healing. Because radiotherapy is one of the most widely used anti-neoplastic treatments for head and neck cancer, reducing the harmful side effects of radiation will greatly impact patient quality of life. Thus, this innovative strategy to reduce the side effects of radiotherapy and improve patient tolerance to treatment is promising for clinical application. In addition, MSCs are attractive candidates for the delivery of anti-tumor reagents or cytokines in human malignancies. MSC treatment will lead to a safer and more effective clinical therapy for tumors and reduce radiotherapy or chemotherapy complications. However, further investigations are needed to establish the long-term safety and effects of MSC transplantation therapy.

## Materials and methods

### Animal experiments

Male mice (C57) were purchased from the Animal Center of Sun Yat-sen University (Guangzhou, China). All the experiments were approved by the Animal Care and Use Committee of Sun Yat-sen University (IACUC-DB-16-1215). Animal models were established as previously described^42^. The tongue was extended out of the mouth and exposed to 16 Gy (a dose of 1.6 Gy/min) of radiation through a 10-mm-diameter hole to establish RIM. Oral ulcers were chemically induced by placing a 3 × 3-mm round filter paper soaked with 70% acetic acid on the buccal mucosa for 30 s. The results are representative of three independent experiments (six animals per group). Detailed information is shown in Supplementary Fig. 1.

### Isolation and characterization of MSCs

MSCs were obtained from healthy donors who provided informed consent. MSCs were isolated from the bone marrow using our previously reported method^13^. Briefly, bone marrow cells were separated by Ficoll-Paque (1.077 g/ml; Amersham Biosciences, Uppsala, Sweden) density gradient centrifugation. MSCs were maintained in growth medium consisting of low-glucose Dulbecco’s modified Eagle’s medium (L-DMEM; HyClone, Logan, UT, USA) and 10% fetal bovine serum (FBS; Gibco, Grand Island, NY, USA). The MSCs were then seeded into T25 cell culture flasks at a density of 1 × 10^5^ cells/ml. The culture-expanded MSCs exhibited surface expression of CD44, CD73, CD90, and CD105 (MSC markers) but not CD34 or CD45 (hematopoietic markers). After the fourth passage, the capacity of MSCs to differentiate into multiple lineages was confirmed by their differentiation to osteoblasts, chondrocytes, or adipocytes, which was performed using previously described methods^13^.

### RNA isolation, reverse transcription, and RT-qPCR

Total RNA was extracted using TRIzol (Invitrogen, Carlsbad, CA, USA) according to the manufacturer’s instructions. Reverse transcription was performed using ReverTra Ace (Toyobo Co, Ltd, Osaka, Japan), and RT-qPCR was performed using SYBR PCR Master Mix (Roche, Indianapolis, IN, USA). Three independent experiments were performed. The signals were detected using a Light Cycler 480 Detection System (Roche, Sweden). The primer sequences are listed in Supplementary Table 1.

### Western blot analysis

Cells and tissues subjected to the indicated treatments were prepared, lysed in radioimmunoprecipitation assay (RIPA) buffer, and centrifuged at 10,000 rpm and 4 °C for 10 min. The proteins were separated by sodium dodecyl sulfate-polyacrylamide gel electrophoresis (SDS-PAGE) and transferred to a poly vinylidene fluoride (PVDF) membrane (Millipore, Bedford, MA, USA). The membrane was then blocked with 5% milk for 1 h, incubated with the appropriate primary antibodies at 4 °C overnight, and then incubated with horseradish peroxidase-conjugated secondary antibodies for 1 h at room temperature. Bands were detected with a chemiluminescence kit (Millipore). The primary and secondary antibodies are listed in Supplementary Table 2.

### Immunofluorescence staining

The samples were embedded in the Tissue-Tek optimum cutting temperature (OCT) compound (Miles Scientific, Naperville, IL, USA) and frozen in a frozen slicer (Leica CM1900, Germany). The sections (5 mm thick) were placed on glass slides (Bio-Optica SpA, Milano, Italy), blocked with PBS-Tween 0.05% plus 0.5% FBS for 30 min, and successively incubated with the appropriate primary antibodies overnight at 4 °C and secondary antibodies for 30 min at room temperature.

### Lentiviral vector construction and infection

Lentiviral vectors expressing the CXCR2 gene were constructed by and purchased from Hanheng Biotechnology (Shanghai, China) (Supplementary Fig. 3A). For lentiviral infection, the optimal multiplicity of infection (MOI) was 50 (50 viral particles for each cell), which was determined in our previous experiments. According to the manufacturer’s instructions, the cells were incubated with the virus overnight, and this solution was then removed and replaced with new complete medium.

### Flow cytometry

The expression levels of CXCR2 and GFP on MSCs were analyzed by flow cytometry (FACScan; Becton Dickinson, San Diego, CA, USA) as previously described^43^. Briefly, the MSCs were incubated for 30 min with the appropriate antibody in the dark at 4 °C and then analyzed by flow cytometry. CXCR- and GFP-positive cells were used for further study. Each analysis was performed with at least three independent experiments. The data were analyzed with FlowJo7.5 (Treestar, Ashland, OR, USA) or Kaluza (Beckman Coulter, Krefeld, Germany) software.

### BLI

BLI of luc is a reliable noninvasive imaging tool used to quantitatively monitor MSC survival and distribution with high sensitivity. Briefly, after anesthetizing the mice via an intraperitoneal injection of 2% pentobarbital (5 μl/g) or inhalation of isoflurane anesthesia, the reporter probe D-luciferin (Invitrogen) was injected into the mouse model (1500 mg/kg), and the tongue was removed from the mouth, placed as close as possible to the detector and imaged for 1 min using a Bruker Xtreme imaging system (Bruker, Karlsruhe, Germany). Signals (photons/second/cm^2^/steradian) from a fixed region of interest (ROI) were evaluated according to the manufacturer’s instructions.

### CCK-8 assay

The CCK-8 assay was performed according to the manufacturer’s instructions (Dojin Laboratories, Kumamoto, Japan). Briefly, 100 µl of cell suspension was dispensed (5000 cells/well) into a 96-well plate. The plate was pre-incubated for 24 h in growth medium (37 °C, 5% CO_2_), and 10 µl of various concentrations of toxicant (H_2_O_2_) were added to the culture medium in the plate. The plate was then incubated for an appropriate length of time in the incubator. CCK-8 solution (10 µl) was added to each well of the plate, and the plate was incubated for 1–4 h in the incubator. The absorbance at 450 nm was measured using a microplate reader (Tecan, Infinite f200 PRO, Switzerland). The relative growth rate (RGR) was calculated using the following formula:

$${\rm{RGR}} \\ = \frac{{{\rm{OD}}\ {\rm{value}}\ \left( {{\rm {experimental}}\ {\rm {group}}} \right) - {\rm {OD}}\ {\rm {value}}\ \left( {{\rm {blank}}\ {\rm {control}}} \right)}}{{{\rm {OD}}\ {\rm {value}}\ \left( {{\rm {negative}}\ {\rm {control}}} \right) - {\rm {OD}}\ {\rm {value}}\ \left( {{\rm {blank}}\ {\rm {control}}} \right)}}.$$

### Migration assays

Migration was assessed using a transwell chamber system with 8-μm-pore filters (PIEP12R48, Millipore). Each upper chamber was loaded with serum-starved MSCs (2 × 10^5^ per well), and each lower chamber was loaded with 500 μl of serum-free medium with or without hCXCL2 (5 ng/ml, Peprotech) or mCXCL2 (50 ng/ml, Peprotech). After a 6- to 12-h incubation at 37 °C in 5% CO_2_, the cells remaining on the upper surfaces were removed with a cotton swab, and the filters were fixed and stained with 0.1% crystal violet. Cells that migrated to the lower surface were counted under a microscope

### In vivo distribution of transplanted MSCs

MSCs^CXCR2^ and MSCs^GFP^ were examined on days 1 and 3 post-injection in oral RIM/CIM models. Cryosections were prepared and counterstained with 1.0 μg/ml DAPI in PBS for 3 min at room temperature in the dark.

### Primary tongue cell culture

Tongue epithelial cells and fibroblasts were prepared and maintained as previously described^44,45^. Briefly, both cell types were isolated from mouse tongue tissue specimens. Tongue epithelial tissues and connective tissues were dissociated with 4% dispase and 4% collagenase I (37 °C for 1 h or 4 °C overnight). The tongue epithelial tissues could then easily be separated from whole tissues with forceps. Tongue epithelial cells were derived from epithelial tissues, and fibroblasts were derived from connective tissues. High-glucose DMEM and DMEM/F12 with 10% FBS were used as the growth media for the primary cultures of epithelial cells and fibroblasts, respectively. The primary cells were plated at a density of 1 × 10^5^ cells per 25-mm culture dish. Third-passage cells were used in the experiments.

### ROS assessment

The levels of total intracellular ROS and mitochondrial ROS were detected using the fluorescent probe CellROX Deep Red (Life Technologies, Carlsbad, CA, USA), and the fluorescence intensity was measured by flow cytometry. In addition, DHE was used to evaluate the in situ formation of O_2_^−^ according to the manufacturer’s instructions. Briefly, tongue sections were incubated with DHE (DHE, 1:1000 dilution, National Diagnostics, Atlanta, GA, USA) for 5 min at 37 °C. The tissue sections were visualized under a fluorescence microscope with a 590-nm wavelength filter.

### Flow cytometry analysis of apoptosis

Primary tongue epithelial cells and fibroblasts were treated with H_2_O_2_ for 6 h and then cultured with or without MSCs for 48 h. The cells were digested, monitored, and then stained for 15 min with Annexin V and PI (Biosci Biotech, Shanghai, China) in binding buffer according to the manufacturer’s instructions. The apoptotic population was assessed by flow cytometry. Annexin V+/PI− cells represent early apoptotic cells, whereas Annexin V+/PI+ cells are late apoptotic cells.

### Statistics

All the data are presented as the means ± SEMs from at least three independent experiments. Comparisons between groups were performed using Student’s *t*-test or one-way analysis of variance (ANOVA) with a Newman–Keuls post hoc comparison. *P* < 0.05 was considered statistically significant. All the statistical analyses were performed with SPSS version 19.0 (SPSS Inc., Chicago, IL, USA).

## Electronic supplementary material

Supplementary Information
