# Supplementary material for: Genetic modification to induce CXCR2 overexpression in mesenchymal stem cells enhances treatment benefits in radiation-induced oral mucositis
Source: Cell Death Dis. 2018 Feb 14;9(2):229. doi: 10.1038/s41419-018-0310-x (PMC5833705; doi:10.1038/s41419-018-0310-x)
Supplement: Supplementary file 1 — Supplementary Information [file 41419_2018_310_MOESM1_ESM.docx]

**Supplementary information**


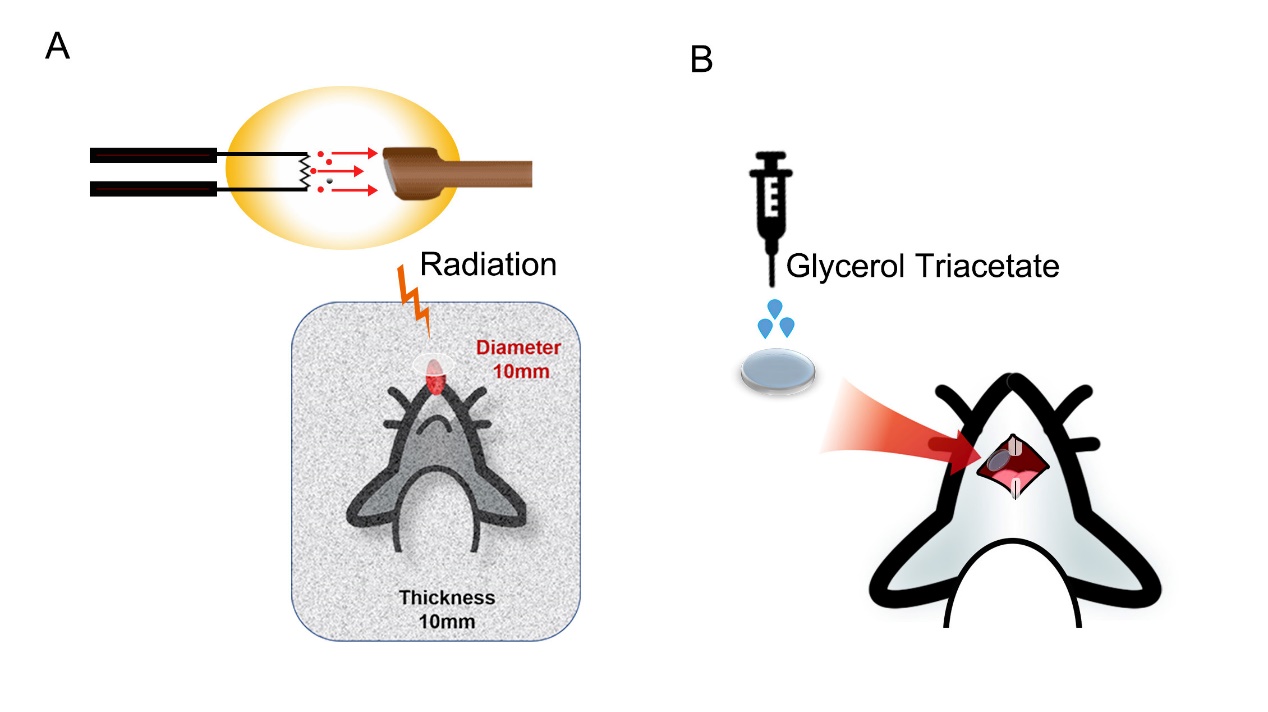


**Supplementary Figure 1. Schematic of the animal models**

(A) The tongue was brought out of the mouth and exposed to 16 Gy (a dose of 1.6 Gy/min) of radiation through a 10-mm-diameter hole.

(B) Chemical-induced mucositis was generated by placing a 3 x 3-mm round filter paper soaked with 70% acetic acid on the buccal mucosa for 30 seconds.


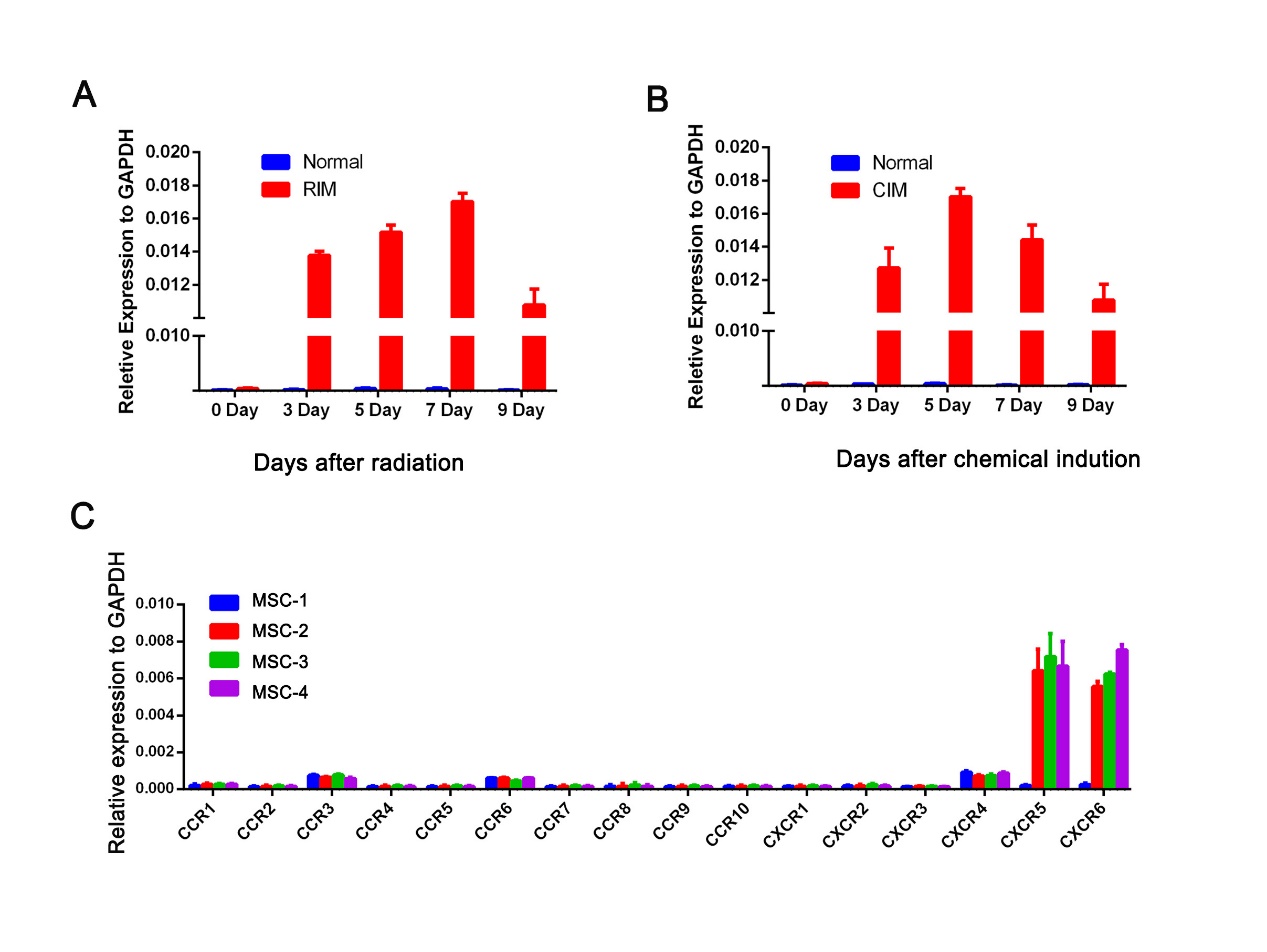


**Supplementary Figure 2. The expression pattern of CXCL2 in mucositis, with low chemokine receptor expression on MSCs**

(A) Levels of the CXCL2 mRNA from day 0 to day 9 post-radiation. The GAPDH mRNA was detected as an internal control. Data are presented as the means ± SEM (n = 6) for each group.

(B) Levels of the CXCL2 mRNA from day 0 to day 9 post-chemical induction. The GAPDH mRNA was detected as an internal control.

(C) Levels of chemokine receptor mRNAs in sixth-passage MSCs from additional four donors. The GAPDH mRNA was detected as an internal control. Data are presented as the means ± SEM (n = 4) for each group.


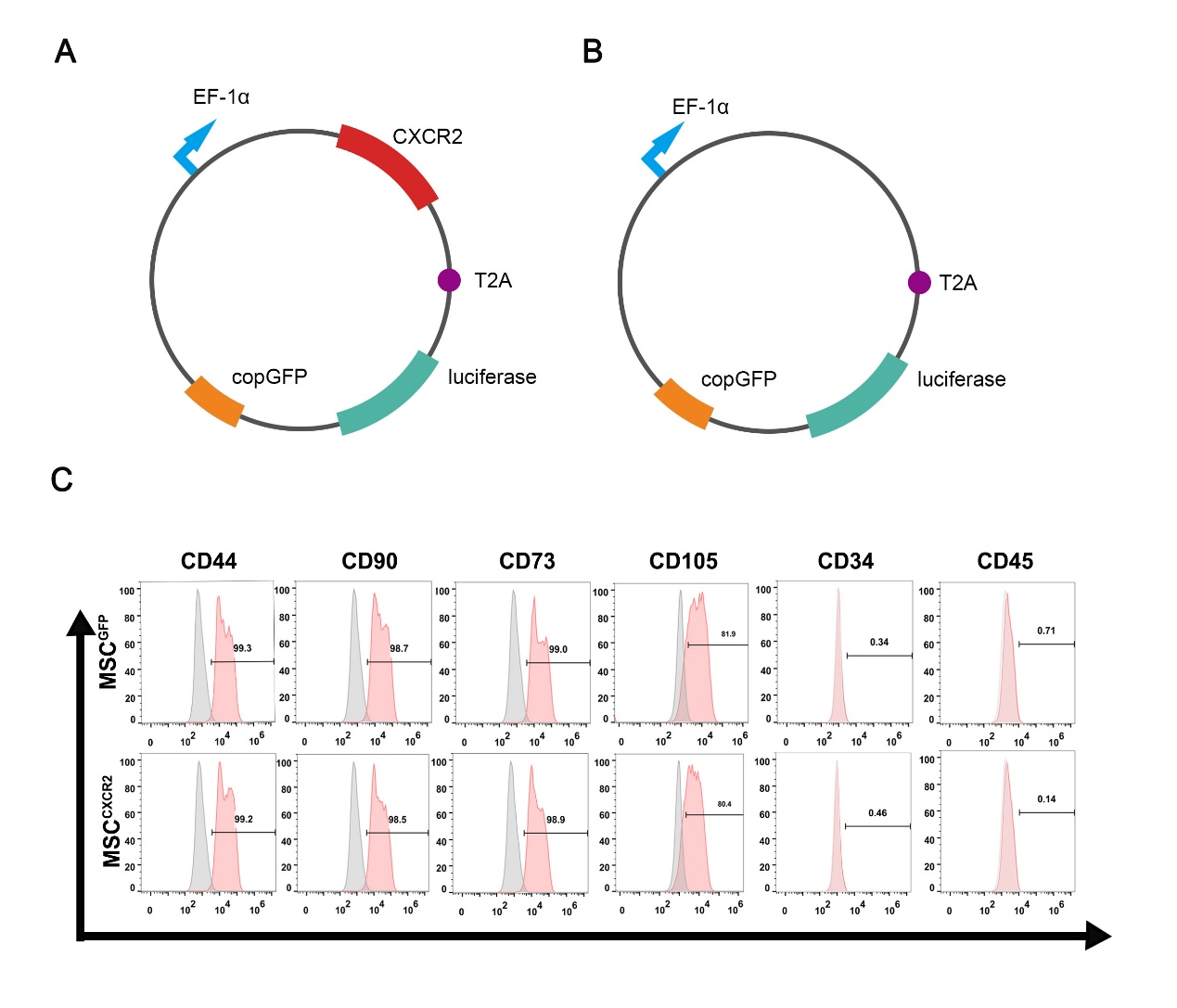


**Supplementary Figure 3. Lentiviral infection to produce CXCR2-overexpressing MSCs do not alter the expression of stem cell markers**

(A) Plasmid constructs. Luc and GFP were inserted after the chemokine receptor CXCR2 under the same promoter.

(B) Plasmid constructs. Luc and GFP were inserted under the same promoter.

(C) Flow cytometry analysis comparing the expression levels of the indicated stem cell surface markers on MSCs^CXCR2^ and MSCs^GFP^. MSCs^CXCR2^ expressed the same pattern of stem cell surface markers as MSCs^GFP^.


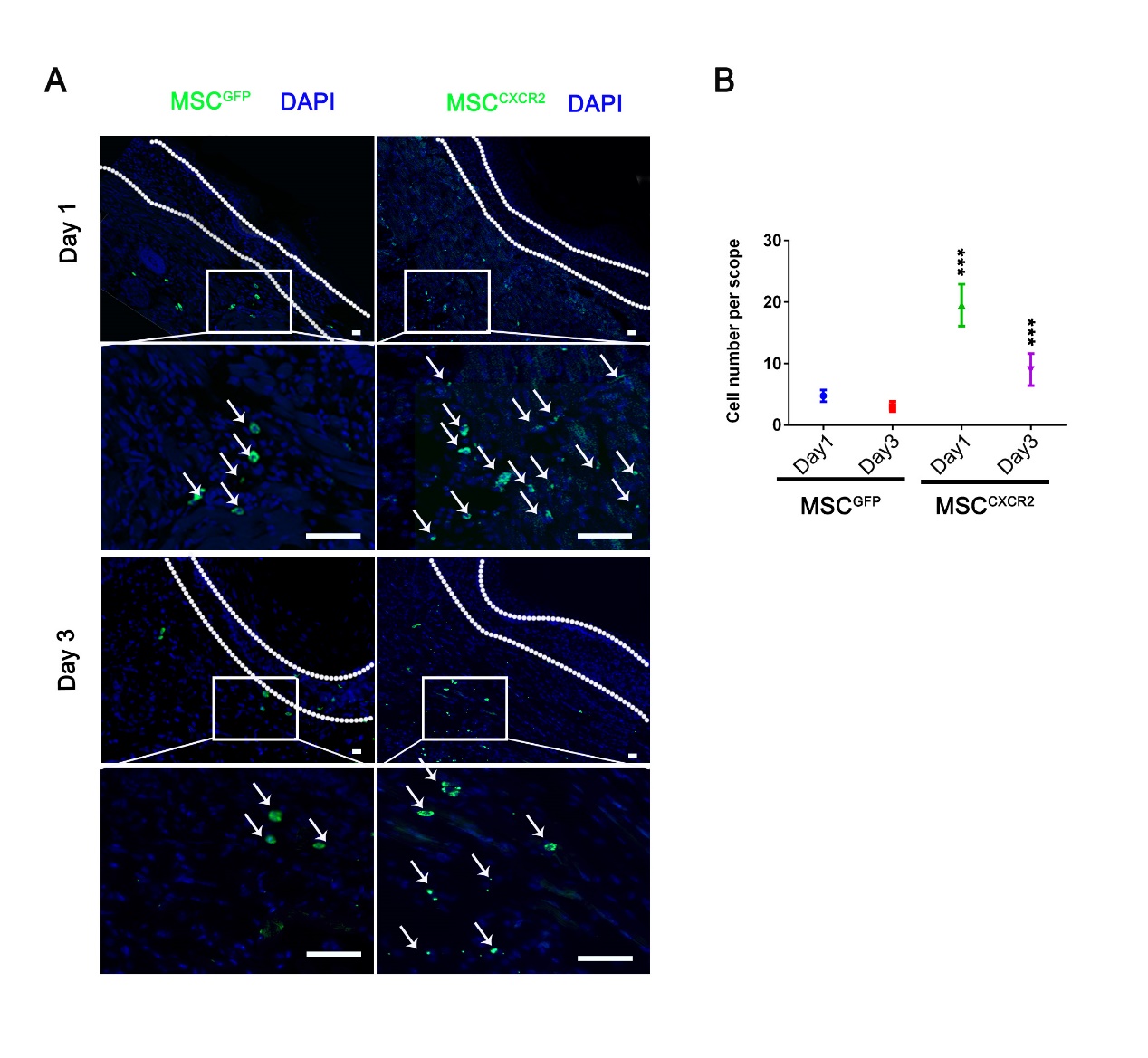


**Supplementary Figure 4. MSCs^CXCR2^ exhibit enhanced migration toward the inflamed mucosa**

(A) MSCs^CXCR2^ and MSCs^GFP^ were examined by immunofluorescence staining, both of which expressed GFP when injected into chemical-induced oral mucositis models on days 1 and 3 post-injection. Signals: GFP, green; DAPI, blue. Scale bar = 100 μm.

(B) GFP-positive cells were quantified in each microscopic field of the mouse tongue. Data are presented as the means ± SEM for each group (n = 6, t-test). Date are representative of three independent experiments. ***P < 0.001 the CIM + MSCs^CXCR2^ group compared with the CIM + MSCs^GFP^ group.


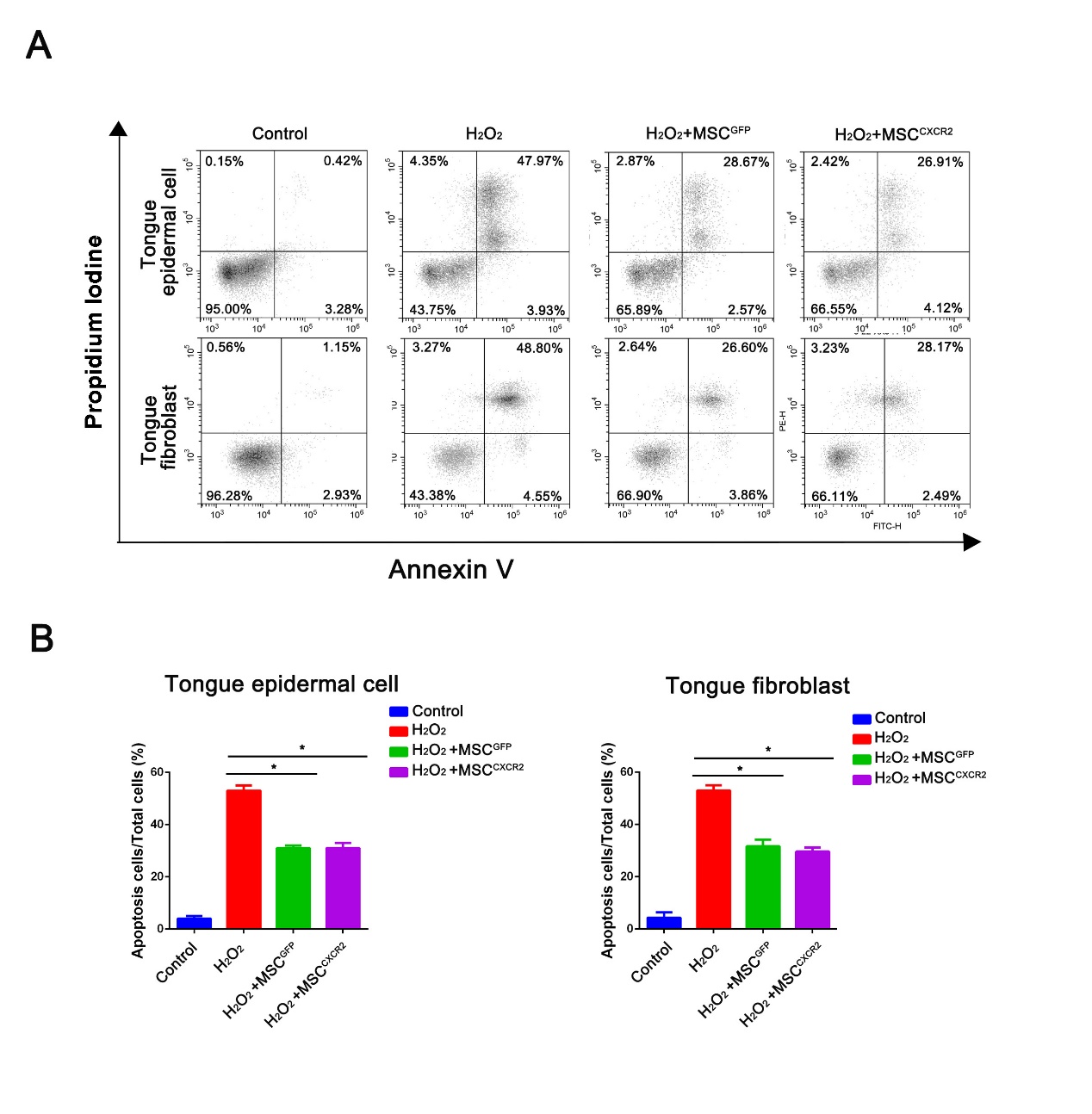


**Supplementary Figure 5. MSCs protect primary tongue cells from oxidative stress-induced apoptosis**

(A) Primary tongue epithelial cells and fibroblasts were treated with H_2_O_2_ for 6 h and

then cultured with or without MSCs^GFP^ or MSCs^CXCR2^ for 48 h. Apoptosis was measured by Annexin V/PI staining and flow cytometry.

(B) Histograms were quantiﬁed to analyze the percentage of Annexin V-positive cells. Data are presented as the means ± SEM (n = 3) for each group (***P < 0.001; t-test).

| **Supplementary Table 1. Primers used to amplify the transcripts in real-time quantitative PCR** | | |
| --- | --- | --- |
| **Gene (human)** | **Sequence (5′ to 3′)** | **Application** |
| **CD44** | **Upper: CTGCCGCTTTGCAGGTGTA**  **Lower: CATTGTGGGCAAGGTGCTATT** | **qRT-PCR** |
| **CD90** | **Upper: ATCGCTCTCCTGCTAACAGTC**  **Lower: CTCGTACTGGATGGGTGAACT** | **qRT-PCR** |
| **CD73** | **Upper: AAGGACTGATCGAGCCACTC**  **Lower: GGAAGTGTATCCAACGATTCCCA** | **qRT-PCR** |
| **CD105**  **CCR1** | **Upper: CGCCAACCACAACATGCAG**  **Lower: GCTCCACGAAGGATGCCAC**  **Upper: CCTGCTGACGATTGACAGGTA**  **Lower: TCTCGTAGGCTTTCGTGAGGA** | **qRT-PCR**  **qRT-PCR** |
| **CCR2** | **Upper: TACGGTGCTCCCTGTCATAAA**  **Lower: TAAGATGAGGACGACCAGCAT** | **qRT-PCR** |
| **CCR3** | **Upper: TGGCATGTGTAAGCTCCTCTC**  **Lower: CCTGTCGATTGTCAGCAGGATTA** | **qRT-PCR** |
| **CCR4**  **CCR5**  **CCR6** | **Upper: AGAAGGCATCAAGGCATTTGG**  **Lower: ACACATCAGTCATGGACCTGAG**  **Upper: GTTGGACCAAGCTATGCAGGT**  **Lower: GCAGAAGCGTTTGGCAATGT**  **Upper: GGCTATTTGTACCGATTGCCT**  **Lower: GATGCCTTTTAGCAACTTGCAC** | **qRT-PCR**  **qRT-PCR**  **qRT-PCR** |
| **CXCR1** | **Upper: CTGACCCAGAAGCGTCACTTG**  **Lower: CCAGGACCTCATAGCAAACTG** | **qRT-PCR** |
| **CXCR2** | **Upper: CCTGTCTTACTTTTCCGAAGGAC**  **Lower: TTGCTGTATTGTTGCCCATGT** | **qRT-PCR** |
| **CXCR3** | **Upper: GGTCATGGCCTACTGCTATGC**  **Lower: CCACGTCTACCCTGCTTTCT** | **qRT-PCR** |
| **CXCR4**  **CXCR5**  **CXCR6**  **GAPDH** | **Upper: ACTACACCGAGGAAATGGGCT**  **Lower: CCCACAATGCCAGTTAAGAAGA**  **Upper: GGTCACCCTACCACATCGTC**  **Lower: GCCATTCAGCTTGCAGGTATTG**  **Upper: GACTATGGGTTCAGCAGTTTCA**  **Lower: GGCTCTGCAACTTATGGTAGAAG**  **Upper: TGTGGGCATCAATGGATTTGG**  **Lower:**  **ACACCATGTATTCCGGGTCAAT** | **qRT-PCR**  **qRT-PCR**  **qRT-PCR**  **qRT-PCR** |

| **Gene (Mouse)** | **Sequence (5′ to 3′)** | **Application** |
| --- | --- | --- |
| **CCL2** | **Upper: TTAAAAACCTGGATCGGAACCAA**  **Lower: GCATTAGCTTCAGATTTACGGGT** | **qRT-PCR** |
| **CCL8** | **Upper: TCTACGCAGTGCTTCTTTGCC**  **Lower: AAGGGGGATCTTCAGCTTTAGTA** | **qRT-PCR** |
| **CCL17**  **CCL19**  **CCL21** | **Upper: TACCATGAGGTCACTTCAGATGC**  **Lower: GCACTCTCGGCCTACATTGG**  **Upper: CCTGGGAACATCGTGAAAGC**  **Lower: TAGTGTGGTGAACACAACAGC**  **Upper: GTGATGGAGGGGGTCAGGA**  **Lower: GGGATGGGACAGCCTAAACT** | **qRT-PCR**  **qRT-PCR**  **qRT-PCR** |
| **CXCL1**  **CXCL2**  **CXCL3**  **CXCL5** | **Upper: TCGAGACCATTTACTGCAACAG**  **Lower: CATTGCCGGTGGAAATTCCTT**  **Upper: CCAACCACCAGGCTACAGG**  **Lower:** **GCGTCACACTCAAGCTCTG**  **Upper: GAAAGGAGGAAGCCCCTCAC**  **Lower: TGGCCAGCCAAGGAATACTG**  **Upper: GTTCCATCTCGCCATTCATGC**  **Lower: GCGGCTATGACTGAGGAAGG** | **qRT-PCR**  **qRT-PCR**  **qRT-PCR**  **qRT-PCR** |
| **CXCL9**  **CXCL10**  **CXCL12**  **GAPDH** | **Upper: GGAGTTCGAGGAACCCTAGTG**  **Lower: GGGATTTGTAGTGGATCGTGC**  **Upper: CCAAGTGCTGCCGTCATTTTC**  **Lower:** **GGCTCGCAGGGATGATTTCAA**  **Upper: TGCATCAGTGACGGTAAACCA**  **Lower: TTCTTCAGCCGTGCAACAATC**  **Upper: AATGGATTTGGACGCATTGGT**  **Lower: TTTGCACTGGTACGTGTTGAT** | **qRT-PCR**  **qRT-PCR**  **qRT-PCR**  **qRT-PCR** |

**Supplementary Table 2. Antibodies used for immunoblotting**

| **MARKER (SPECIES)** | **DILUTION** | **DISTRIBUTOR/SOURCE**  **(CATALOG NUMBER)** |
| --- | --- | --- |
| **Primary antibody:** |  |  |
| **CXCL2 rabbit mAb**  **GFP Rabbit mAb**  **α-Tubulin (11H10) Rabbit mAb**  **ERK 1/2 antibody**  **p-ERK (Thr202/Tyr204) antibody**  **Akt (C67E7) rabbit mAb**  **p-Akt rabbit mAb** | **1:10000(WB)**  **1:200(IF)**  **1:1000**  **1:2000**  **1:1000**  **1:1000**  **1:1000**  **1:1000** | **R&D (AF-452-SP )**  **CST (2956)**  **CST (2125)**  **CST (9102)**  **CST (9101s)**  **CST (4691s)**  **CST (4046s)** |
| **Secondary antibody:**  **Anti-mouse IgG HRP-linked Ab**  **Anti-rabbit IgG HRP-linked Ab**  **Anti-rabbit IgG** **(Fluor® 594 Conjugate)**  **Antibody for Flow cytometry**  **PE-CXCR2 Antibody**  **APC-CD44 Antibody**  **FITC-CD105 Antibody**  **APC-CD73 Antibody**  **APC-CD90 Antibody**  **PE-CD34 Antibody**  **APC-CD45 Antibody** | **1:5000**  **1:5000**  **1:500**  **1:100**  **1:100**  **1:100**  **1:100**  **1:100**  **1:100**  **1:100** | **CST (7076)**  **CST (7074)**  **CST (8889)**  **Biolengend (2565691)**  **Biolengend (103010)**  **Biolengend (** **323203 )**  **Biolengend (344005)**  **Biolengend (328113)**  **Biolengend (343506)**  **Biolengend (103211)** |
